# The formin Drosophila homologue of Diaphanous2 (Diaph2) controls microtubule dynamics in colorectal cancer cells independent of its FH2-domain

**DOI:** 10.1038/s41598-019-41731-y

**Published:** 2019-03-29

**Authors:** Saskia S. Grueb, Stefanie Muhs, Yannes Popp, Sebastian Schmitt, Matthias Geyer, Yuan-Na Lin, Sabine Windhorst

**Affiliations:** 1 Department of Biochemistry and Signal Transduction, University Medical Center Hamburg-Eppendorf Martinistrasse 52, D-20246 Hamburg, Germany; 2 Institute of Structural Biology, University of Bonn, Sigmund-Freud-Str. 25, D-53127 Bonn, Germany; 3 Department of General, Visceral and Thoracic Surgery, University Medical Center Hamburg-Eppendorf Martinistrasse 52, 52 D-20246 Hamburg, Germany

## Abstract

In this study, we analyzed the functional role of the formin Drosophila Homologue of Diaphanous2 (Diaph2) in colorectal cancer cells. We show that stable down-regulation of Diaph2 expression in HT29 cells decreased chromosome alignment and the velocity of chromosome movement during M-phase, thus reducing the proliferation rate and colony formation. In interphase cells, Diaph2 was diffusely distributed in the cytosol, while in metaphase cells the protein was located to spindle microtubules (MTs). Diaph2-depletion increased the concentration of stable spindle MTs, showing that the formin is required to control spindle MT-dynamics. Our cellular data indicate that Diaph2-controls spindle MT-dynamics independent of Cdc42 activity and our *in vitro* results reveal that bacterially produced full-length (FL) Diaph2 strongly altered MT-dynamics in absence of Cdc42, where its actin-nucleating activity is auto-inhibited. FL-Diaph2 mediates a 10-fold increase in MT-polymerization compared to the Diaph2-FH2-domain. Interestingly, a Diaph2-mutant lacking the FH2-domain (ΔFH2) increased MT-polymerization to a similar extent as the FH2-domain, indicating the existence of a second MT-binding domain. However, in contrast to FL-Diaph2 and the FH2-domain, ΔFH2 did not alter the density of taxol-stabilized MTs. Thus, the FH2-domain and the second Diaph2-binding domain appear to control MT-dynamics by different mechanisms. In summary, our data indicate that Diaph2 controls M-phase progression under basal conditions by regulating spindle MT-dynamics. In addition, a region outside of the canonical MT-regulating FH2-domain is involved in Diaph2-mediated control of MT-dynamics.

## Introduction

Drosophila homologue of Diaphanous (Diaph)-proteins belong to the family of formins, which stimulate both actin nucleation and microtubule (MT) stabilization^1–3^. The conserved FH1 domains bind profilin/G-actin and the conserved dimeric FH2-domains bind to barbed ends of actin filaments protecting them from capping. In addition, the FH1/FH2-domains of different formins directly bind to microtubules (MTs) *in vitro*^1^. Cheng *et al*.^2^ revealed that the bacterially produced FH1/FH2-domains of mDia3 (the mouse homologue of human Diaph2) directly bind to and stabilize MTs *in vitro*. In cells, however, Diaph2 is associated with MTs as a complex with the +TIP proteins End-binding Protein 1 (EB1) and Adenomatous-polyposis-coli (APC)^2^ that stabilize MTs^2^.

In the absence of extracellular stimuli, the actin nucleating activity of Diaph formins is auto-inhibited through interaction of the C-terminal Diaphanous Autoregulatory Domain (DAD) with the N-terminal Diaphanous Inhibitory Domain (DID). Binding of Rho-GTPases to the N-terminus attenuates this auto-inhibition and stimulates actin nucleation, albeit to a lower extent than the C-terminus lacking the DAD domain^4,5^. Whether MT-binding of Diaph-proteins is regulated through auto-inhibition is not clear but it is proposed that the Rho family GTPase Cdc42 controls MT-attachment to kinetochores by activating mDia3^6^.

In mammalian cells three different isoforms of Diaph have been identified: Diaph1–3. The isoforms are highly similar and it is unclear why cells express three different Diaph forms. Over the past few years, Diaph1 has been recognized as a key molecule controlling a variety of cellular and morphogenetic functions in physiological, as well as pathological settings^7,8^. Moreover, up-regulated Diaph1 expression has been detected in many cancer types^9^. Recently, we found a specific up-regulation of Diaph1 in patient samples from colorectal carcinomas and showed a positive correlation between Diaph1 expression and the presence of colon cancer metastasis^10^. Further mechanistic studies revealed that stable down-regulation of DIAPH1 in colon carcinoma cells nearly completely blocked metastasis in SCID mice by regulating MT-dependent cellular adhesion^11^.

In contrast to Diaph1, the relevance of Diaph2 in cancer progression has been less investigated. A potentially novel kinetochore function for Diaph2, however, was identified in HeLa cells previously^12^. An siRNA-induced Diaph2 knock down resulted in chromosome misalignment as well as in impaired spindle attachment to chromosomes, indicating that Diaph2 may be important for spindle MT dynamics. A related study confirmed the essential role for Diaph2 for chromosome alignment and revealed that the isolated FH1/FH2-domains of Diaph2 stabilize MTs in an Aurora-B dependent manner^6^. From these data the authors concluded that Diaph2 controls chromosome alignment by stabilizing spindle MTs. Since failures in chromosome alignment can result in unequal distribution of chromosomes and thus in chromosomal instability (CIN)^13^, it seems crucial to investigate if Diaph2 is involved in the control of CIN in colon cancer.

In this study, we addressed the functional role of human Diaph2 for progression of colorectal carcinoma cells and its potential function in chromosome alignment, metaphase progression and MT-modification.

## Material and Methods

### Cell lines

HT29 cells were purchased from American Type tissue Culture Collection through European Tissue Culture Collection. The cell line was cultured in Dulbecco’s modified Eagle’s medium (DMEM, Gibco), supplemented with 10% (v/v) fetal calf serum (FCS), 100 μg/ml streptomycin, and 100 units/ml penicillin. Cells were cultured at 37 °C in a humidified incubator with 5% CO_2_.

### Stable knock down of Diaph2

Stable knock down of Diaph2 expression in HT29 cells was performed as described in Windhorst *et al*.^14^. Five different vectors (Mission shRNA Plasmid DNA pLKO.1 shRNA) from Sigma, coding for shRNA against Diaph2 were tested. After selection with puromycin (2 µg/ml) Diaph2 k.d.1 and Diaph2 k.d.4 showed 90% (Diaph2 k.d.1) and 95% (Diaph2 k.d.4) reduced DIAPH2 expression, respectively. Cells stably expressing scrambled shRNA served as control. All *in vitro* data shown here were reproduced with both Diaph2 k.d.1 and Diaph2 k.d.4.

### Quantification of mRNA levels

For RNA extraction the NucleoSpin RNA Kit (MACHEREY-NAGEL) was used. Total RNA concentration was determined photometrically using Nanodrop (Peqlab), and RNA quality was assessed by electrophoresis on a denaturing agarose gel. Using the SuperScript III Reverse Transcriptase (Invitrogen), 1 μg of total RNA was reverse transcribed. Quantitative RT-PCR analysis was carried out on the LightCycler 2.0 Instrument (Roche) using the Mastermix LightCycler FastStart DNA Master SYBR Green I (Roche). Samples were analyzed in duplicate and averaged using the Light Cycler Software 3.5 (Roche). Data were analyzed based on the ΔΔCt method using HT29 Diaph2 control cells as a reference sample. All primers (Table S1) were design to amplify products between 90 and 200 bp, and exon-spanning primers were used to avoid DNA amplification.

### Measurement of proliferation and apoptosis

Proliferation was examined by image analysis, measuring the confluence of the cells, using the software IncuCyte Zoom 2016B (Essen BioScience). For measurement of apoptosis the Caspase 3/7 –Assay was evaluated by the IncuCyte system. Therefore, the Caspase-3/7 Red Apoptosis Assay Reagent (IncuCyte by Essen BioScience) was applied to the cells. In apoptotic cells the caspases cleave and activate the dye. The red stained apoptotic cells are detected by an internal fluorescence reader of the IncuCyte system and are evaluated by the IncuCyte software IncuCyte Zoom 2016B (Essen BioScience).

### Analysis of colony formation potential

To figure out the potential of the cells to survive and to form colonies from single cells, 1000 cells diluted in 2 ml cell culture medium (DMEM with 10% FCS) were applied to one well of a 6-well plate. The cells were incubated for 10 days, washed with PBS and fixed with 4% paraformaldehyde solution containing 4% sucrose. After washing the cells with water, they were stained with 500 μl Giemsa Azur-Eosine-Methylblue solution (1:10) for 10 min. The cells were washed again with water, dried at room temperature and the number of colonies was counted.

### Determination of chromosomal alignment

To visualize chromosomal alignment, the cells were synchronized with a double thymidine block^15^. For this purpose the cells were incubated 16 h in media containing 2 mM thymidine, which blocks DNA replication. Thereafter, the replication block was removed by releasing the cells into fresh media. After further incubation for 9 h the cells were treated again with 2 mM thymidine for 15 h to enrich mitotic cells. After incubation with fresh medium (release) for 6 h the cells were fixed with 4% paraformaldehyde/4% sucrose, stained with DAPI (4′,6-Diamidin-2-phenylindol) and an antibody against ß-tubulin (Sigma-Aldrich Cat# T4026), coupled with an Alexa-fluor-conjugated 568 antibody (Jackson ImmunoResearch). The slides were analyzed by the Keyence Microscope; chromosomes and MTs as well as merged micrographs were documented.

### Chromosome preparation

Cells were incubated with Democolchicine (0.3 µg/ml) to induce metaphase arrest. After 6 h incubation with Democolchicine the cells were harvested with trypsin and resuspended in PBS, engorged with 75 mM KCl-solution and fixed with a mixture of methanol and acetic acid (3:1). Finally the cells were spotted on glass trays and stained with Giemsa solution. The chromosomes were documented by the Keyence Microscope.

### Measurement of Cdc42 activity by PAK pull down and stimulation of Cdc42 activity

The Rac/Cdc42 (p21) binding domain (PBD) of the human p21 activated kinase 1 protein (PAK) protein binds specifically to GTP-bound Rac and Cdc42 proteins. Here, the PAK-PBD domain was expressed in *E. coli* as GST fusion protein and coupled to glutathione sepharose beads. To isolate GTP-bound Rac and Cdc42 from HT29 cells, the cells were seeded to 10 cm dishes and grown to 70% confluence. The cells were washed twice with ice cold phosphate saline (PBS) containing 0.5 mM CaCl_2_ and 1 mM MgCl_2_. 1 ml lysis buffer (50 mM Tris/HCl, pH 7.5; 1 mM EDTA, 150 mM NaCl, 1% (v/v) NP-40, 1 mM PMSF) was added, the cells scraped with a cell scraper and frozen at −80 °C to improve lysis. After thawing, the suspension was vortexed twice, centrifuged for 15 min at 13.000 × g and 4 °C. Protein concentration was determined by the Bradford assay. Supernatants with equal protein concentrations were incubated with 20 µl of the PAK-PBD beads for 30 min at 4 °C. The sample was centrifuged for 2 min at 4 °C for 2.500 rpm, the supernatant was discarded and the beads were washed 3 times with lysis buffer. Binding of GTP-bound Cdc42 was analyzed by Western blotting.

In order to stimulate Cdc42 activity, the Cdc42/Rac1 GTPase inhibitor ML141 (Merck, Calbiochem # 217708) was used (see also Hong *et al*.^16^). Since GTPases hydrolyze GTP, in presence of the GTPase inhibitor ML141, Cdc42/Rac1 remain in their GTP-bound, thus, active state. HT29 cells were incubated with 20 µM or 40 µM ML141 for 2 h or 4 h, or with DMSO as vehicle control, respectively. Thereafter, the level of GTP-bound Cdc42 was analyzed by the PAK assay (see above).

### Measurement of duration of mitosis by labeling histone 2B (H2B)

Cells were transfected with a vector encoding for pH2B_EYFP using the Lipofectamine LTX with Plus Reagent (Thermo Fisher Scientific). 24 h after transfection, cells were imaged for 24 h every 5 min using the Visitron Spinning disk-TIRF, including an environmental chamber with temperature, humidity and CO_2_ control. Analysis of nuclei was performed with the Fiji-imaging software (NIH National Institutes of Health). Finally, the time from the beginning until the end of mitosis was determined.

### Western blot analysis

Western blot analysis was performed by using a standard procedure. In brief, equal amounts of protein (30 μg) were loaded to SDS PAGE, which was blotted to nitrocellulose (NC). The membranes were blocked 30 min at room temperature with 5% milk powder in Tris-buffered saline and Tween 20 (TBST). After this the membranes were incubated with the respective antibodies in 5% milk/TBST solution overnight at 4 °C. The antibodies used were: rabbit anti-Diaph2 (Sigma-Aldrich Cat# HPA005647), rabbit anti-ß-tubulin (Sigma-Aldrich Cat# T4026), mouse anti-actin (Sigma Cat# A 2066), rabbit anti-Hsc70 (Santa Cruz Biotechnology Cat# sc-7298), rabbit anti-detyrosinated ß-tubulin (abcam Cat# ab48389), mouse anti-Cdc42 (BD Bioscience Cat# 610928). Secondary antibodies against mouse (1:10.000; abcam Cat# ab205719) or rabbit (1:10.000; abcam Cat# ab205718) were diluted in TBST and incubation was performed for 1 h at room temperature. After visualization by chemiluminescence reagent (Amersham ECL Prime Western Blotting Detection Reagent, GE Healthcare Bio-Sciences) and ImageQuant LAS 4000 (GE Healthcare), band intensities were quantified by ImageJ (NIH National Institutes of Health) and calculated as percent intensity of the specific control sample.

### Immunocytology

2.5 × 10^5^ cells were seeded to µ-slide-8-well (Ibidi) coated with poly-L-lysine, grown to 50% confluence, washed with PBS and fixed with pre-warmed (37 °C) 4% paraformaldehyde/DMEM/4% FCS for 15 min at 37 °C. After incubation for 15 min with 0.1% Triton-X-100/PBS at room temperature, the primary antibodies (see above) were used in a dilution of 1:200 in PBS containing 4% FCS and incubated at RT for 1 h. Secondary antibodies conjugated with Alexa-fluor 488 or 568 (Jackson ImmunoResearch) were used in a dilution of 1:2000 in PBS containing 4% FCS and incubation was performed for 1 h at room temperature. Nuclei were stained by incubating the cells with DAPI 1:2000 in PBS for 5 min at room temperature. After washing with PBS, stained cells were analyzed by fluorescence microscopy. For co-localization studies of Diaph2 with tubulin, the confocal microscope TCS SP8 (Leica) was used. IMARIS Image Analysis Software (Bitplane) was used for further evaluation.

For immunofluorescence analysis of detyrosinated microtubules, 5 × 10^4^ cells were seeded on µ-slide-8-well (Ibidi) and incubated for 24 h. Thereafter, cells were fixed with ice cold methanol at −20 °C for 5 min. All washing steps were performed with 0.05% Triton X/PBS. Blocking was performed with 1% BSA in 0.05% Triton X/PBS for 1 h. Anti-detyrosinated α-tubulin antibody (ab48389, abcam) was diluted 1: 200 in blocking solution. Cells were incubated with primary antibody solution for 1 h. Secondary Alexa-fluor-conjugated 488 antibody (Jackson ImmunoResearch), was diluted 1:200 in 0.05% Triton X/PBS. Analysis was performed at TCS SP5 (Leica) and ImageJ/Fiji was used for further evaluation.

### Recombinant expression of Diaph2, FH2 and Diaph2-ΔFH2 in bacteria

The cDNA coding for Diaph2 was sub-cloned into the pGEM-T Easy vector (Promega) and cDNAs transferred into the vector pGEX-6P-2A using XhoI and NotI restriction site. Additionally, the cDNA was mutated by QuikChange® mutagenesis to delete the nucleotides coding for amino acids 624 to 1049 (ΔFH2). The FH2-domain (aa 624–1049) was cloned into pGEX-4T vector.

The full-length protein (FL), FH2-domain or ΔFH2 deleted Diaph2 proteins were expressed in BL21(DE3)pLysS *E. coli*. The bacteria were incubated at 37 °C to OD600 = 0.8 and then treated with 0.7 mM Isopropyl-β-D-thiogalactopyranosid (IPTG) over night at 20 °C. Cells were harvested (4500 × g, 10 min 4 °C) and washed with PBS. Thereafter, the pellet was resuspended in lysis buffer (PBS, 10% glycerol, 1 mM EDTA, 1 mM DTT, 5 mg/mL lysozyme, 0.5 mM PMSF und 1 × protease inhibitor (Roche)) and sonicated three times for 60 sec. Lysates were centrifuged at 48.000 × g (20.000 rpm), 45 min at 4 °C. The supernatant was filtered with a 0.45 µM pore filtropur filter (Sarstedt). Glutathione sepharose-beads (GE Healthcare) were washed three times with running buffer (20 mM HEPES, 150 mM KCl, 10% glycerol, 1 mM DTT and protease inhibitor cocktail (Roche)). Lysates and the same amount of running buffer were applied to the beads and shaken for two hours at RT. Beads were washed 3 times with lysis buffer. Elution was performed with elution buffer (lysis buffer containing 30 mM glutathione, pH 8). Beads were shaken for 10 min and the supernatants were collected as elution fraction. The elution was repeated twice. The protein amount of the elution fractions and the remaining protein amount of the beads were quantified by SDS-Page stained with Roti Blue quick (Roth). Beads and soluble proteins were stored at 4 °C to maintain activity of the proteins.

### MT-pull down assay

To analyze binding of Diaph2-proteins to MTs, tubulin (Cytoskeleton, # T240) diluted in general tubulin (GT)-buffer (80 mM PIPES pH 6.9, 2 mM MgCl_2_ and 0.5 mM EGTA) to a concentration of 10 mg/ml (0.2 mM) was incubated with 1 mM GTP for 5 h at 37 °C to produce MTs. The DIAPH proteins were centrifuged for 15 min at 15.000 × g at 4 °C and 49 µl of the supernatant, containing 4 µM protein, was incubated with 1 µl MTs (see above) for 5 min RT. To analyze binding of Diaph2 proteins to MTs, the samples were centrifuged for 30 min at 100.000 × g at 25 °C. Diaph2 protein without MTs were also centrifuged for 30 min at 100.000 ×g at 25 °C to control non-specific pelleting.

### MT-bundling

1 µM of rhodamine-conjugated, taxol-treated microtubules (cytoskeleton, #TL590M) solution in general tubulin (GT)-buffer (80 mM PIPES pH 6.9, 2 mM MgCl_2_ and 0.5 mM EGTA) containing 20 µM taxol and 1 mM GTP was incubated for 5 h at 37 °C to produce MT filaments. 0.1 µM Diaph2 proteins were incubated with 1 µM of pre-polymerized microtubules for 30 min at 37 °C in low protein binding micro-tubes (Sarstedt # 72.706.600). The mixture was centrifuged for 10 min at 4000 × g. Supernatant and pellet were solved in SDS-sample buffer and analyzed by Roti Blue quick (Roth) stained SDS-Page.

### Analysis of MT polymerization

Shelanski *et al*.^17^ and Lee *et al*.^18^ demonstrated that light is scattered at 340 nm by microtubules proportional to the growth of polymerized microtubules. Results, thus, represent nucleation, growth and steady state equilibrium of microtubules. To analyze the effect of Diaph2 proteins on MT-polymerization, the proteins were concentrated using Vivaspin 500 centrifugal concentrate (Sigma-Aldrich) and re-buffered in GT-buffer containing 10% glycerol and 1 mM GTP. For measurement of MT-polymerization, 2 µM tubulin and 0.2 µM Diaph2 proteins in 100 µl GT-buffer containing 10% glycerol and 1 mM GTP were applied to 96-well plates (pre-warmed to 37 °C) and measured at 340 nm with the Tecan Infinite M200 plate reader for 2 hours at 37 °C.

### MT polymerization imaging

This experiment was performed as described in Heinz *et al*.^19^. For cold-induced depolymerization, 0.01 µM Diaph2 proteins were incubated with 0.1 µM of rhodamine-conjugated, taxol-treated microtubules (cytoskeleton, #TL590M) for 20 min at 4 °C and vortexed before imaging.

### Statistical analysis

Statistical comparisons was performed with GraphPad Prism version 3.03 or R version 3.5.2. Normalized, normally distributed values in classified groups were statistically analyzed using One-Way ANOVA and subsequent Bonferroni’s multiple comparison test or the random incept model in R. For analysis of Western blot protein level and mRNA level with less than 4 values (no normal distribution test is possible) a normal distribution was assumed. Not normally distributed values were analyzed with the Kruskal-Wallis statistics and further compared with Dunn’s multiple comparison test. P-Values indicating significant differences between groups are: *p < 0.05, **p < 0.01 and ***p < 0.001.

## Results

### Diaph2 controls chromosome alignment in colorectal carcinoma cells

To examine the functional role of Diaph2 in colorectal carcinoma cells, the protein was stably depleted in cells with high endogenous Diaph2 expression (HT29) using a lentiviral shRNA approach. The success of this manipulation was controlled by real time PCR and Western blotting (Figure S1). In HT29 cells, the level of the Diaph2 protein was decreased by 90% (k.d.1 = knock down 1) and by 95% (k.d.4 = knock down 4), as compared to scrambled shRNA control cells. For all further experiments, we compared scrambled control cells with Diaph2 k.d.1 and k.d.4 cells.

**Figure 1:**
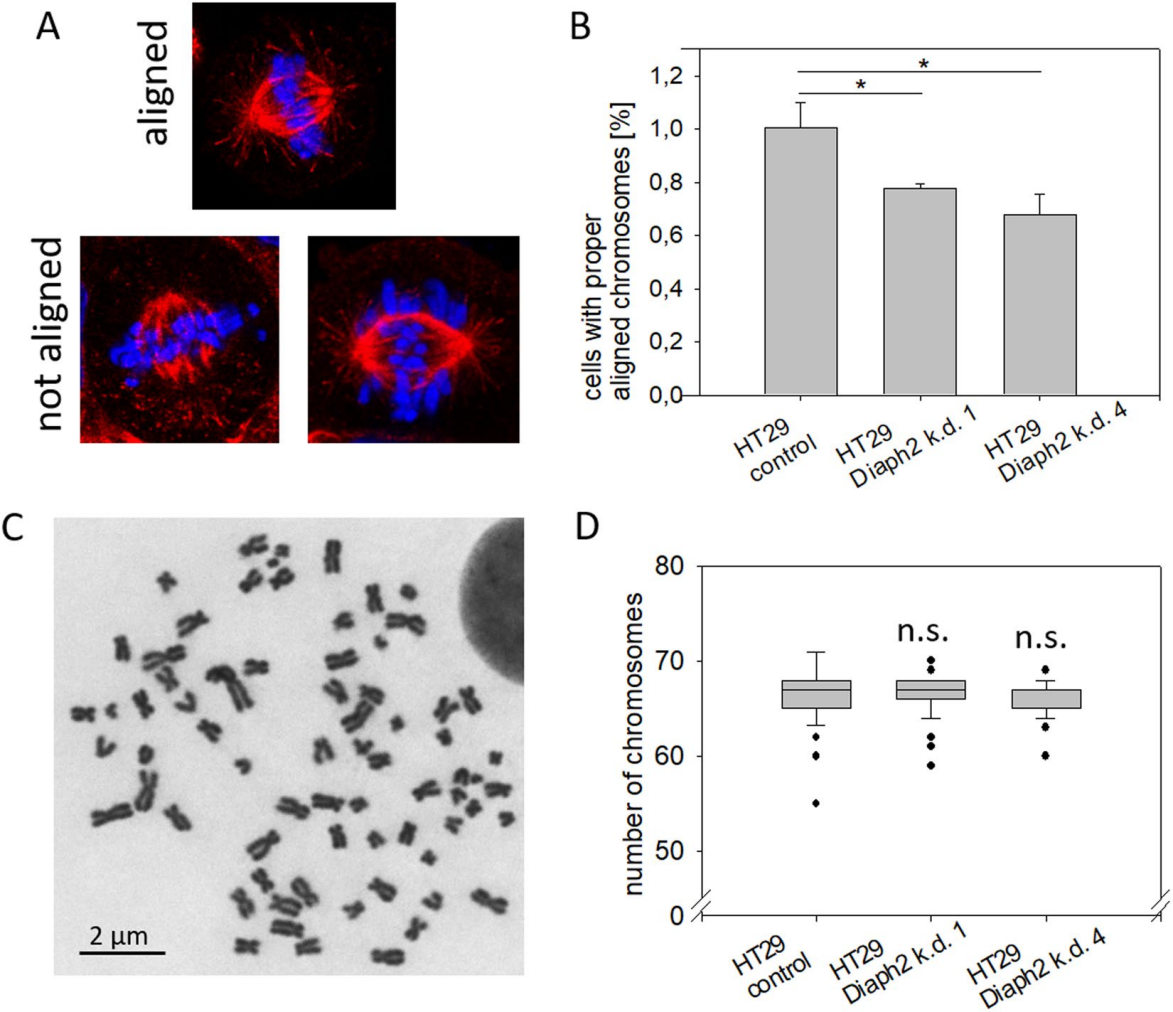
Diaph2 controls chromosome alignment. (**A**) Cells were synchronized in M phase and chromosomes were stained by DAPI (blue) and spindle fibers by a β-tubulin-specific antibody (red). Shown are representative cells with aligned or not aligned chromosomes. (**B**) The number of cells with proper aligned or not aligned chromosomes were counted. The percentage of cells with proper aligned chromosomes was determined and the value obtained from control cells was set to one. Shown are mean values + SD of 30 cells from three independent experiments. Values were normally distributed and evaluated with One-Way ANOVA and Bonferroni’s multiple comparison test, *p < 0.05 (**C**). Chromosomes were prepared from metaphase cells and stained with Giemsa solution. Shown is one representative preparation from control cells. (**D**) The number of chromosomes of 30 control or Diaph2-depleted cells from three independent experiments was determined. Shown are mean values + SD. Values were not normally distributed and were tested with Kruskal-Wallis statistic and Dunn’s Multiple Comparison test.

In Hela cells, Diaph2 is required for proper chromosome alignment by controlling the dynamics of kinetochore spindles^6^. In order to analyze whether this was also the case in colorectal carcinoma cells, modified HT29 cells were synchronized in M-phase and chromosomes and MTs were stained with DAPI and with a β−tubulin specific antibody, respectively. Thereafter, correct chromosome alignment was analyzed by fluorescence microscopy. In Fig. 1A, examples of aligned and not aligned chromosomes are shown. The number of cells with aligned or with not aligned chromosomes was determined, the percentage of cells with aligned chromosomes was calculated and normalized to control cells (Fig. 1B). This analysis revealed that depletion of Diaph2 reduced the number of cells with aligned chromosomes by 20% (k.d.1) and 30% (k.d.4), indicating that Diaph2 is indeed involved in the control of chromosome alignment in colorectal carcinoma cells.

Next, we analyzed if chromosomal misalignment in Diaph2-depleted HT29 cells leads to unequal distribution of chromosomes after cellular division. For this purpose, the number of chromosomes was analyzed in control and in Diaph2-manipulated cells (see example in Fig. 1C). It is of note to mention that HT29 cells are chromosomally instable^20^, therefore also in control cells the chromosome number is altered, as compared to chromosomal stable colorectal cells, i.e. HCT116 (see Figure S2). However, depletion of Diaph2 in HT29 cells did not significantly alter the number of chromosomes (Fig. 1D). Together, these data show that depletion of Diaph2 results in chromosomal misalignment but has no significant effect on distribution of chromosomes.

### Effect of Diaph2 depletion on chromosome kinetics

**Figure 2:**
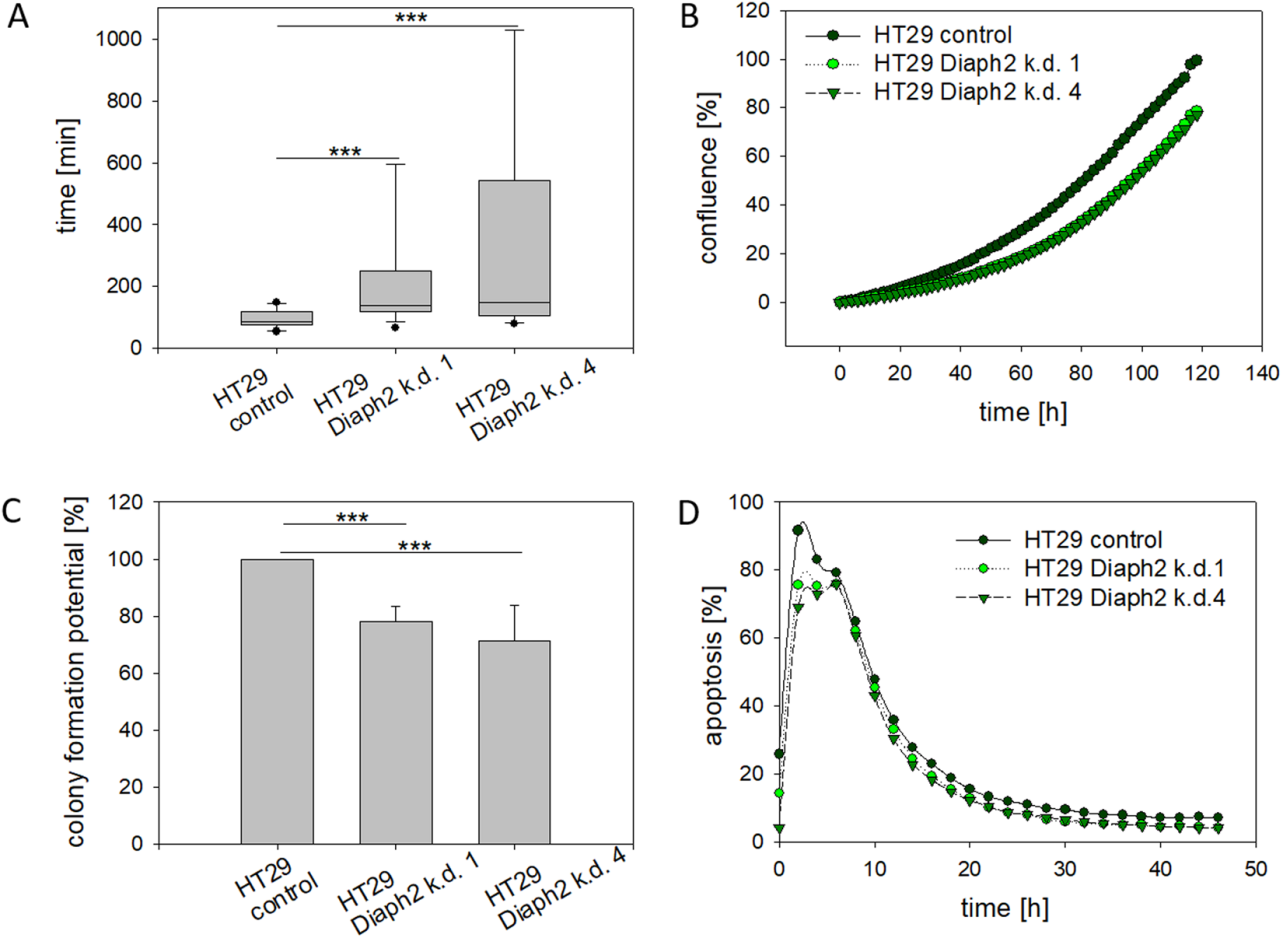
Diaph2 controls chromosome velocity, proliferation and colony formation. (**A**) HT29 cells were transfected with a vector encoding for pH2B-EYFP and 12 h after transfection pH2B_EYFP fluorescence was monitored by live cell imaging (see also Figure S3). The duration of M-phase was calculated from 30 cells from three independent experiments. Mean values ± SD were determined. Values were normally distributed and evaluated with an unpaired t-Test ***P < 0.0001. (**B**) Cellular proliferation was analyzed by live cell imaging using the IncuCyte System. Shown are mean values of three independent experiments. (**C**) 1000 cells were seeded to 6-well plates and after 10 days of incubation, the colonies were stained with Giemsa solution and counted by hand. Shown are mean values + SD of 6 wells, which have been evaluated in each of 3 different experiments. Single values as raw data were used to calculate significance by the random incept model with R-statistic analysis program, ***p < 0.001. **(D**) The cells were incubated with Caspase-3/7 Red Apoptosis Assay Reagent (IncuCyte) to label apoptotic cells and percent of apoptotic cells were determined by using the IncuCyte System. Shown are mean values of three independent experiments.

Though Diaph2 did not affect distribution of chromosomes, it was likely that the protein is required for chromosome kinetics because this process depends on precisely regulated MT-dynamics^21^. To address this question, HT29 control and Diaph2-depleted cells were transfected with pH2B_EYFP and chromosome kinetics in M-phase were analyzed by live cell imaging (Figure S3). From these data we calculated duration of mitosis and found significantly prolonged M-phase progression in Diaph2-depleted cells as compared to control (Figs 2A and S3). In addition, evaluation of the live cell imaging images revealed that Diaph2-depleted cells also exhibited a higher number of not aligned chromosomes in anaphase (Fig. S3B).

To analyze if prolonged M-phase progression affects proliferation or colony formation, we compared these cellular processes between control and Diaph2-depleted cells. Our data revealed that both proliferation and colony formation were reduced by 20% in Diaph2-depleted cells (Fig. 2B,C). However, apoptosis was not altered after depletion of Diaph2 (Fig. 2D).

In summary, depletion of Diaph2 resulted in chromosomal misalignment with subsequent prolonged M-phase progression and decreased proliferation and colony formation.

### Diaph2 localizes to spindle-MTs and alters spindle MT-dynamics

In Hela cells, Diaph2 controls chromosome alignment by stabilizing kinetochore-MT-attachment^2^. If in HT29 cells Diaph2 regulates chromosome alignment by controlling dynamics of spindle MTs, the protein should be located to spindle MTs in M-phase. To address this assumption, localization of Diaph2 in HT29 cells was analyzed using Diaph2-specific Alexa-fluor488-conjugated (green) and β-tubulin-specific Alexa-fluor568-conjugated (red) antibodies.

**Figure 3:**
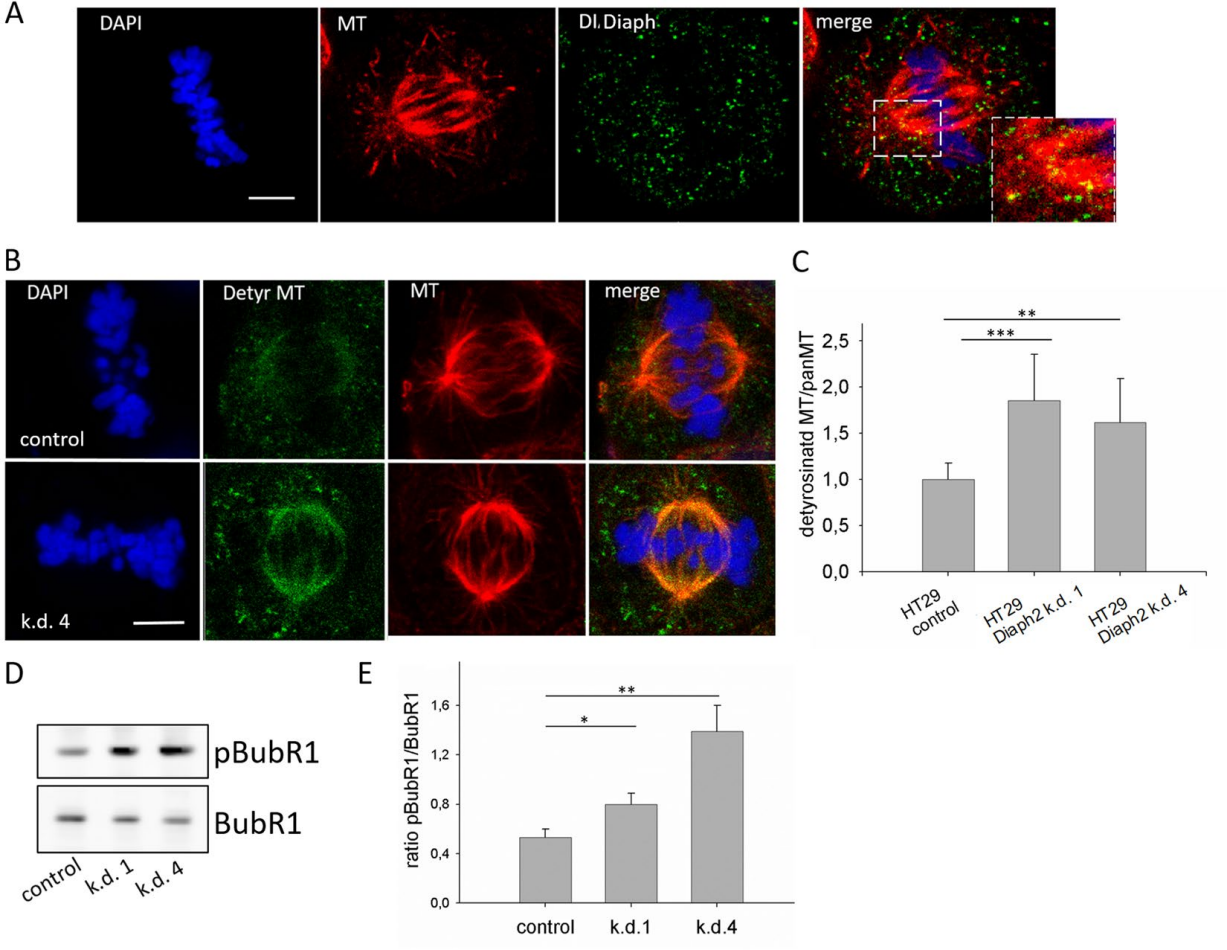
Diaph2 is localized to spindle MTs and controls spindle dynamics. (**A**) Control HT29 cells were stained with an Alexa-fluor-488-coupled antibody against Diaph2 (green), with an antibody against β-tubulin (red) and with DAPI to stain the chromosomes (blue). (**B**) M-phase cells were stained with an Alexa-fluor-488-coupled antibody against detyrosinated MTs (green) and with an Alexa-fluor-568-coupled antibody against β-tubulin (red), chromosomes were stained with DAPI (blue). (**C**) Signals derived from detyrosinated MTs and from β-tubulin (panMT) were quantified by ImageJ from values of 10 different cells from one representative experiment out of 4 and the ratio of detyrosinated MTs to panMT was calculated. Shown are mean values + SD. Values were normally distributed and evaluated with One-Way ANOVA and Bonferroni’s multiple comparison test **p < 0.01 and ***p < 0.001. (**D**) The protein level as well as the concentration of phospho-BubR1 Tyr680 (pBubR1) were analyzed in control and Diaph2-depleted cells by Western blotting. (**E**) Band intensities of four different signals were analyzed and the ratio of pBubR1/BubR1 was calculated. Shown are mean values + SD. Normal distribution of the band intensity values was assumed, evaluated with One-Way ANOVA and Bonferroni’s multiple comparison test, *p < 0.05 and **p < 0.01.

In interphase cells, Diaph2 was diffusely distributed in clusters in the cytoplasm, but showed no clear co-localization with MTs (Figure S4A). However, Diaph2 co-localized with spindle MTs (see magnification in the right panel of Fig. 3A and also specific control in Fig. S4B) but, different from Hela cells^5^, in HT29 cells Diaph2 did not co-localize with the kinetochore but with MT-populations near the spindle pole (Fig. 3A). To further validate this result, the co-localization index between Diaph2 and MTs was analyzed in 3D-images of HT29 cells using the IMARIS software (Fig. S4C,D). This analysis revealed a co-localization between Diaph2 and spindle MTs of 43.65%, confirming the localization of Diaph2 to spindle-MT populations.

In order to analyze if Diaph2 controls MT-dynamics, lysates of control and Diaph2-depleted cells were analyzed by Western blotting employing an antibody against detyrosinated (thus stabilized) MTs (detyrMT) and, for normalization, an antibody against cellular ß-tubulin (panMT). Quantification of band intensities of detyrMT/panMT, however, revealed no significant differences between control and Diaph2-depleted cells (Fig. S5). Since Diaph2 was distinctly localized to spindle MTs (Fig. 3A), it was likely that the protein did not control cellular MT dynamics in general but only that of spindle MTs. Based on this consideration, we stained metaphase cells with antibodies directed against β-tubulin or detyrMTs (Fig. 3B,C). Calculation of the ratio between fluorescence signals of detyrMTs/MTs revealed that in Diaph2 depleted cells the population of stable spindle MTs was increased 1.5 to 2-fold.

Together with the impaired chromosome alignment in Diaph2 depleted cells, this finding indicates that reduction of Diaph2 expression resulted in defective attachment of spindle fibers to the kinetochore. Since BubR1 is phosphorylated in response to defects in spindle attachment to the chromosomes^22^, we analyzed the phospho-Tyr680 level of BubR1 (pBubR1). Western blot analysis of phosphorylated BubR1 relative to the BubR1 protein level revealed that the pBubR1 concentrations increased in lysates derived from Diaph2-depleted cells as compared to the control sample (Fig. 3D,E).

In conclusion our data strongly indicate that Diaph2 controls chromosome-alignment by regulating the stabilization of spindle-MTs.

### Effects of Cdc42 stimulation and Diaph2-depletion on MT-dynamics in HT29 cells

Yasuda *et al*.^6^ revealed that in Hela cells inhibition and enhancement of Cdc42 activity causes chromosome misalignment and identified mDia3 (Diaph2) as down-stream target of Cdc42. Since knock down of Diaph2 had a similar effect on chromosome misalignment as inhibition of Cdc42, the authors concluded that Diaph2 controls chromosome alignment in a Cdc42-dependent manner.

**Figure 4:**
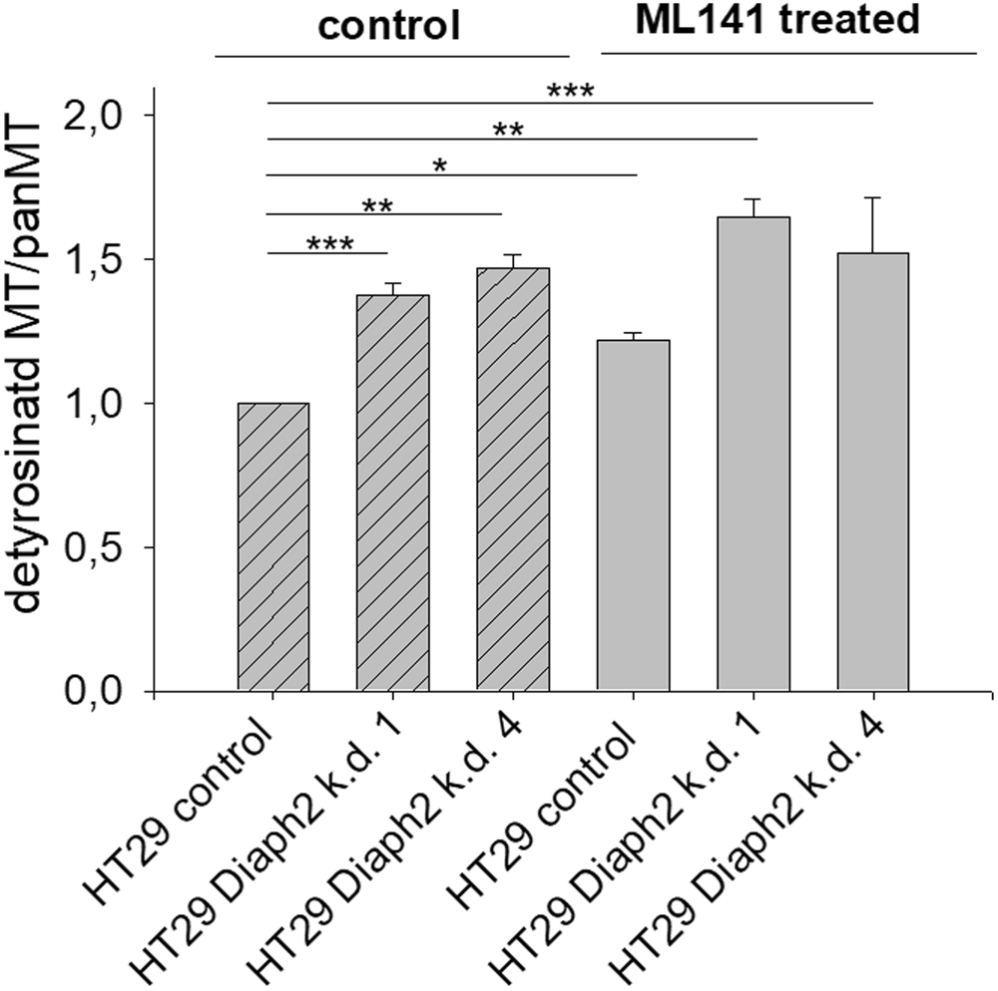
Combined effects of Cdc42 activity and Diaph2 depletion on MT-dynamics. HT29 control and Diaph2-depleted cells were treated with 20 µM ML141 for 2 h, DMSO served as vehicle control. Thereafter, the cells were fixed and stained with antibodies against β-tubulin and detyrMTs, coupled with Alexa-fluor568 or Alexa-fluor488, respectively. The fluorescence intensity derived from these antibodies was analyzed by ImageJ from 10 different cells from one representative experiment out of 4 and the ratio between detyrMTs to MTs was calculated. Shown are mean values + SD. Values were normally distributed, evaluated with One-Way ANOVA and Bonferroni’s multiple comparison test, *p < 0.05, **p < 0.01 and ***p < 0.001.

In order to analyze a potential involvement of Cdc42 activity in Diaph2-controlled MT-dynamics of HT29 cells, we increased the level of active Cdc42 by incubating non-stimulated HT29 control and Diaph2-depleted cells with low concentrations of the Cdc42-GTPase inhibitor ML141 (20 µM for 2 h, see Fig. S6). These cells as well as DMSO-treated control cells were stained with antibodies against β-tubulin and detyrMTs (see Fig. 3B), fluorescence signals were analyzed and the ratio of detyrMTs/MTs was calculated. Again, we found that depletion of Diaph2 resulted in increased MT-stability, confirming our data depicted in Fig. 3C. Interestingly, also Cdc42 activation by ML141 increased MT-stability, independent of the presence (control cells) or absence of Diaph2 (Diaph2-depleted cells) (Fig. 4). This result does not indicate that in HT29 cells Diaph2 controls MT-dynamics in a Cdc42 dependent manner (for details, see discussion below).

### Effect of Diaph2 on MT-polymerization

The observed increase in MT-stability after depletion of Diaph2 in non-stimulated HT29 cells, led us to the hypothesis that also the non-GTPase-bound form of Diaph2 regulates MT-dynamics. For this purpose, full-length (FL) Diaph2, as well as the FH2-domain known to bind to MTs^23^, were expressed in bacteria as GST-fusion proteins and purified by glutathione affinity chromatography.

**Figure 5:**
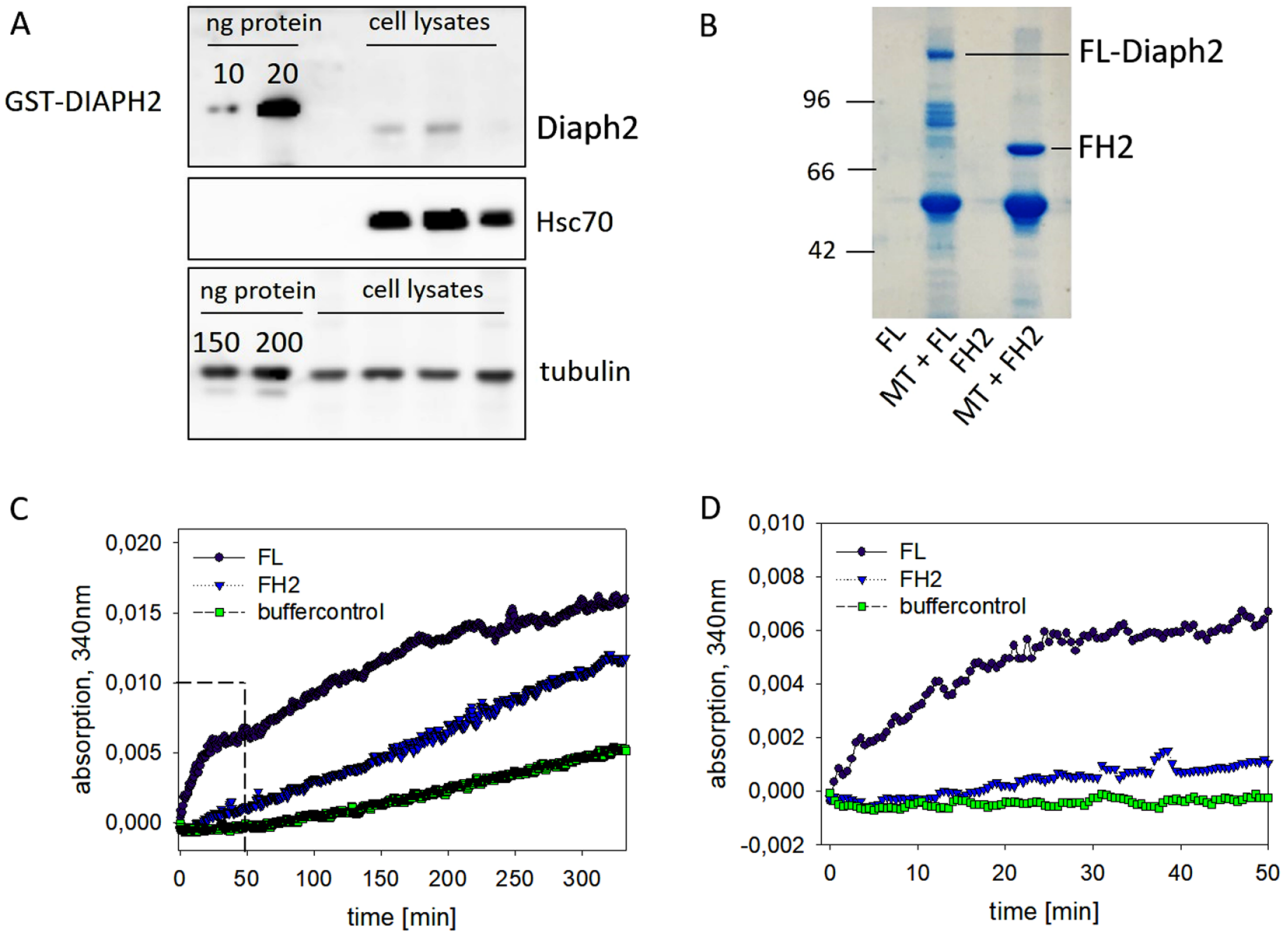
The full-length form of Diaph2 strongly stimulates MT-polymerization. (**A**) Purified tubulin and Diaph2 as well as cell lysates from 2 × HT29 control cells and 1 × Diaph2 k.d.1-cells to detect Diaph2 and from 4 × HT29 control cells to detect tubulin were analyzed by Western blotting, employing antibodies against Diaph2 or β-tubulin, respectively. Hsc70 served as loading control. Band intensities were analyzed by ImageJ and the ratio of MT to Diaph2 was determined to calculate the cellular MT to Diaph2 ratio. (**B**) Purified MTs and full length (FL)-Diaph2 or MTs and the FH2-domain were pulled down by ultracentrifugation. The pellets containing MTs and bound proteins were analyzed by SDS-PAGE. In addition, potential pelleting of FL-Diaph2 or the FH2-domain without MTs were analyzed. (**C**) Polymerization of non-labeled and non-taxol treated MTs was measured in a Tecan reader at 340 nm. Shown is one representative experiment out of three. The right panel shows MT-polymerization during the first 50 minutes of measurement.

Prior to testing the effect of FL-Diaph2 on MT-dynamics by reconstitution assays, the cellular MT to Diaph2 ratio was determined in HT29 cell lysates by Western blotting, employing purified Diaph2 and tubulin as standards (Fig. 5A). Similar band intensities derived from standards and cell lysates were employed to calculate the respective protein concentration per µg total protein. This evaluation revealed a tubulin to Diaph2 ratio of 10 to 1, which was employed in all cell-free *in vitro* assays.

First, we analyzed whether FL-Diaph2, whose actin-nucleating activity is auto-inhibited (Fig. S7), binds to MTs. Therefore, MTs and potentially bound proteins were enriched by ultracentrifugation with the FH2-domain serving as control. SDS-PAGE analysis revealed that both the FH2 and the FL-Diaph2 protein were pulled-down together with MTs (Fig. 5B), indicating that FL-Diaph2 binds to MTs.

Next, we analyzed the effect of Diaph2 on MT-polymerization using a turbidity assay. It is important to note, that when measuring the effect of Diaph2 on MT-polymerization no taxol was added, because taxol itself increases MT-polymerization as well as bundling of MTs^24^. Since we did not detect differences between GST and buffer (Fig. S8), we used buffer as control. Intriguingly, we found that FL-Diaph2 increased the rate of MT-polymerization not only in comparison to the control but also in comparison to the FH2-domain (Fig. 5C). The FH2-domain increased the rate of MT-polymerization 4-fold and FL-Diaph2 even 40-fold as compared to the control. Furthermore, the rate of MT-polymerization immediately increased after addition of FL-DIAHP2 while the increasing effect of the FH2-domain on MT-polymerization first occurred 17 min after incubation (Fig. 5D).

To our knowledge, this is the first time showing that a formin binds to MTs in its auto-inhibited form and, more importantly, increases the velocity of MT-polymerization to a higher extent than the FH2-domain alone.

### Diaph2 increases MT-density

**Figure 6:**
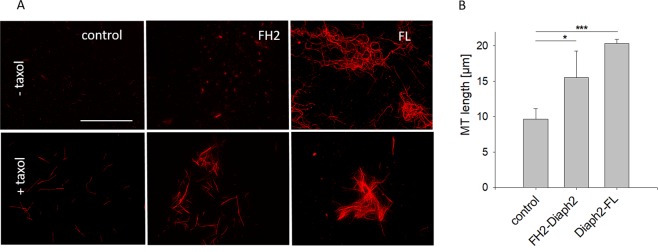
FL-Diaph2 increases the density of MTs. (**A**) Non-treated (-taxol) or taxol-treated (+taxol) rhodamine labeled MTs were incubated with FL-Diaph2 or with the FH2-domain of Diaph2, applied to chamber slides and analyzed by fluorescence microscopy. Scale bar represents 50 µm. (**B**) Length of taxol treated MTs was analyzed. Shown are mean values + SD from 10 different images obtained from one representative experiment out of three. Values were normally distributed and evaluated with One-Way ANOVA and Bonferroni’s multiple comparison test *p < 0.05 and ***p < 0.001.

The result that the FL-length form of Diaph2 had a much stronger effect on MT-polymerization than the FH2-domain alone led us to analyze if MT density is also altered in the presence of FL-Diaph2. For this analysis rhodamine-conjugated MTs were employed, enabling to visualize the length and the density of MTs. This experiment was performed in absence of taxol (Fig. 6A, upper panel) as well as in presence of taxol (Fig. 6A, lower panel).

In absence of taxol, no microtubules were formed in the control approach and in presence of the FH2-domain only short MTs were detectable. Incubation of rhodamine-conjugated MTs with FL-Diaph2, however, resulted in formation of long MTs. In presence of taxol bundled MTs were formed, also in the control sample. Addition of either, the FH2-domain or FL-Diaph2, increased MT density as compared to the control and both Diaph2 forms significantly increased the length of MTs (Fig. 6B). However, in this case, no differences between the FH2 domain and FL-Diaph2 were observed, indicating that taxol treatment attenuates the strong effect of FL-Diaph2 on MT-polymerization.

In summary, our analysis with rhodamine conjugated MTs confirms that FL-Diaph2 promotes formation of MTs.

### Diaph2 increases MT-polymerization independently of its FH2-domain

**Figure 7:**
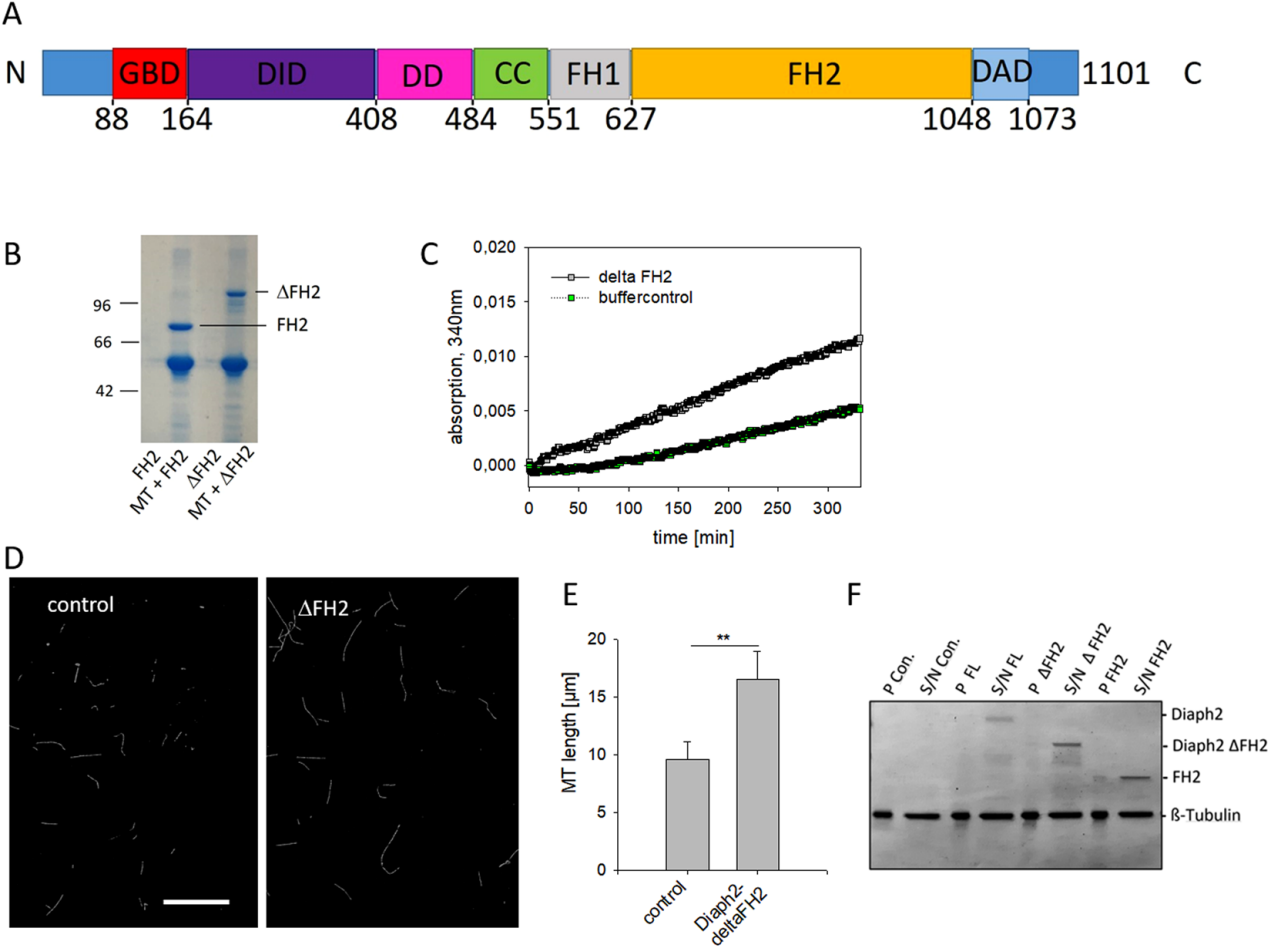
Diaph2 controls MT-dynamics independent of its FH2-domain. (**A**) Cartoon of Diaph2 domains. GBD: GTPase Binding Domain, DID: Diaphanous Inhibitory Domain, DD: Dimerization Domain, CC: Coiled-Coiled domain, FH1: Formin Homology domain 1, FH2: Formin Homology domain 2, DAD: Diaphanous Auto-regulatory Domain (DAD). See also Goode and Eck (2007)^5^. (**B**) MTs and the FH2-domain or MTs and the ΔFH2 mutant were pulled down by ultracentrifugation. The pellets containing MTs and bound proteins were analyzed by SDS-PAGE. In addition, potential pelleting of the FH2-domain or the ΔFH2 mutant without MTs was analyzed. (**C**) Polymerization of non-labeled and non-taxol treated MTs was measured in a Tecan reader at 340 nm. Shown is one representative experiment out of three. (**D**) Rhodamine labeled, taxol-treated MTs were incubated with Diaph2-ΔFH2, applied to chamber slides and analyzed by fluorescence microscopy. Scale bar represents 20 µm. (**E**) Length of taxol treated MTs was analyzed. Shown are mean values + SD from 10 different images from one representative experiment out of three. Values were normally distributed in two groups and evaluated with an unpaired T-Test **p < 0.01. (**F**) MTs in presence or absence of Diaph2 proteins were pulled down by low centrifugation. Pellets (P) and supernatants (S/N) were analyzed by Coomassie-stained SDS-PAGE.

As the effect of FL-Diaph2 on MT-polymerization was stronger than that of the FH2-domain alone (see Figs 5C and 6A upper panel), it was likely that Diaph2 can affect MT-polymerization also independently of its FH2-domain. To test this assumption, a GST-Diaph2 fusion protein with deleted FH2-domain (aa 627–1048, see Fig. 7A) was produced. This Diaph2 mutant was named ΔFH2-Diaph2.

First, we confirmed that ΔFH2-DIAHP2 binds to MTs by the centrifugation-based MT-pull-down assay (Fig. 7B), followed by analysis of the effect of ΔFH2-DIAHP2 on MT-polymerization. We found that ΔFH2-DIAHP2 increased MT-polymerization 6-fold (Fig. 7C), compared to the control. Analysis of taxol-treated, rhodamine-conjugated MTs revealed that MT-length but not MT-density was increased in presence of ΔFH2-DIAHP2 relative to the control sample (Fig. 7D,E). Thus, it seems that a second MT-binding domain of Diaph2 outside of its FH2-domain increases MT-polymerization by a mechanism different from that of the FH2-domain.

Finally, we analyzed if the Diaph2 proteins bundle MTs. For better comparison with the results shown in Figs 6 and 7D, rhodamine-conjugated, taxol treated MTs were used. These MTs were pulled down by low centrifugation and pellets (MT bundles) and supernatants S/N (MTs) were analyzed by Coomassie-stained SDS-PAGE (Fig. 7F). However, the band intensities of MTs in the pellet and the supernatant fractions were not different in presence or in absence of Diaph2 proteins. Thus, at low concentrations Diaph2 does not induce formation of stable MTs bundles.

In summary, our data show that FL-Diaph2 promotes MT-polymerization independent of the FH2-domain, indicating the existence of a second MT-binding domain outside of the FH2-domain.

### Effect of Diaph2 proteins on cold-induced MT depolymerization

**Figure 8:**
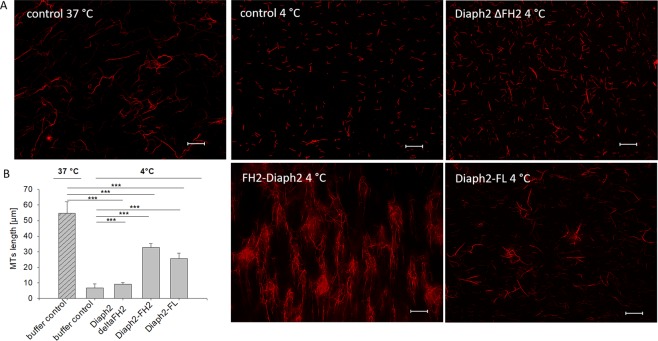
Effect of DIAPH proteins on cold-induced depolymerization of MTs. (**A**) Rhodamine labeled, taxol-treated MTs were incubated at 37 °C and formation of MTs was controlled by fluorescence microscopy. Thereafter, the Diaph2 proteins were added (right panels) and incubation was continued at 4 °C for 20 min. Scale bar represents 20 µm. (**B**) Length of MTs was analyzed. Shown are mean values of 10 different images from one representative experiment out of three. Shown are mean values + SD. Values were normally distributed. They were evaluated with One-Way ANOVA and Bonferroni’s multiple comparison test ***p < 0.001.

Since FH1/FH2-domains of Diaph2 have already been shown to stabilize MTs used at high concentrations^23^, we next analyzed if this was also the case for FL-Diaph2 and ΔFH2- Diaph2 used at an MT to Diaph2 ratio of 10:1. Taxol-treated rhodamine-conjugated MTs were polymerized in absence of Diaph2 proteins and formation of MTs was controlled by fluorescence microscopy (Fig. 8A, left panel). Thereafter, MTs were incubated with Diaph2-proteins for 5 min at RT or remained untreated (control). To destabilize MTs, they were incubated for 20 min at 4 °C (Fig. 8A, right panels). As shown in Fig. 8B, the length of control and DIAHP2-treated MTs strongly decreased after incubation at 4 °C. However, in presence of FL-Diaph2 and the FH2 domain the density as well as length (Fig. 8B) of MTs were increased relative to the control sample.

These result shows that FL-Diaph2 and the FH2 domain attenuate cold-induced MT-depolymerization while ΔFH2-Diaph2 had no effect. Thus, it seems that the FH2 domain mediates the Diaph2 MT-stabilizing effect.

## Discussion

In this study, we analyzed the functional role of Diaph2 in the control of spindle MT-dynamics of colon cancer HT29 cells. It is well-described that precise attachment of kinetochore spindle MTs to the chromosomes is essential for proper chromosome alignment in M-phase^25,26^. Formation of spindle MTs starts by nucleating and elongating MTs from the spindle poles towards the chromosomes. This directed polymerization is controlled by many different MT-associated proteins, among which the kinesins, the dyneins, end binding proteins (i.e. EB1) and further MT-associated proteins promoting polymerization, play essential roles^27–29^. The final attachment of the growing kinetochore MTs to the centromeres of chromosomes is mediated by over 100 kinetochore proteins (the kinetochore), binding as a large protein complex to CENP-A^25,30^. The precise polymerization directed towards the chromosomes as well as binding of MTs to the kinetochore is essential for chromosome alignment in metaphase. Failures of MT-attachment to the kinetochore mostly lead to chromosome misalignments and to unequal distribution of chromosomes to the daughter cells after cellular division (CIN)^13,31^. However, M-phase checkpoint proteins, such as Aurora-B or BubR1 recognize failures in MT-attachment to kinetochores. These proteins then phosphorylate kinetochore substrates, resulting in detachment of MTs from kinetochores until the failure has been repaired. If repair is not possible, the cells undergo apoptosis or become chromosomal instable (CIN)^31^.

Some years ago Diaph2 has been identified as MT-associated protein involved in the regulation of spindle-MT dynamics. Yasuda *et al*. and Cheng *et al*.^2,6^ showed that depletion of Diaph2 in Hela cells resulted in chromosome misalignment due to failures in attachment of spindle-MTs to the kinetochore. Yasuda *et al*.^6^ found Diaph2 to be located at the kinetochore and Cheng *et al*.^2^ revealed an EB1-dependency of Diaph2-controlled spindle MT-dynamics. Furthermore, they showed that the MT-modifying activity of Diaph2 is negatively controlled by Aurora-B, while Yasuda *et al*.^6^ postulated Diaph2 as a down-stream target of Cdc42 (see discussion below).

Based on these data, we examined if also in colon cancer cells Diaph2 is essential for proper chromosome alignment and spindle MT-dynamics. This analysis indeed revealed that depletion of Diaph2 resulted in reduced chromosome alignment in HT29 cells. Since the number of chromosomes was not significantly different between control and Diaph2 depleted cells, Diaph2 does not seem to play a major role in CIN. However, we cannot rule out that Diaph2 depletion slightly affects the chromosome number, which would not be detectable in the chromosomal instable cell line HT29. Our result, showing an increased number of misaligned chromosomes in anaphase, points in this direction.

On the other hand, our data show that depletion of Diaph2 significantly attenuated chromosome kinetics in M-phase, resulting in slowed M-phase progression and thus in a lower proliferation rate. The observation that Diaph2 localizes to spindle MTs and controls spindle MT-dynamics, strongly indicates that Diaph2 controls chromosome kinetics by regulating spindle dynamics. Increased phosphorylation of the checkpoint protein BubR1, which is phosphorylated in response to failures in spindle attachment to the chromosomes^22^, confirms this conclusion. We thus presume that the decreased proliferation rate of Diaph2-depleted cells results from M-phase delay to repair defective attachment of spindle fibers to the chromosomes.

It is important to note that our experiments were performed with non-stimulated cells, a condition where the actin nucleating activity of Diaph2 is auto-inhibited^5^. However, our data show that also under these condition Diaph2 controls MT-dynamics, indicating that the MT-modifying activity of Diaph2 is not auto-inhibited. This finding is in contrast to the hypothesis of Yasuda *et al*.^6^, assuming that the effect of mDia3 (Diaph2) on spindle MT-dynamics depends on Cdc42 stimulation. This conclusion is based on their finding that among the three mDia isoforms only mDia3 binds to GTP-bound Cdc42. Furthermore, they revealed that inhibition and enhancement of Cdc42 activity as well as knock down of mDia3 (also in non-stimulated cells) caused chromosomal misalignment and loss of MT-attachment to chromosomes. However, these result do not reveal that mDia3-mediated control of MT-dynamics depends on Cdc42 activity, but these processes could have occurred in parallel, independent of each other. Our data strongly indicate that at least in HT29 cells this is the case. Although we also found that activated Cdc42 alters MT-dynamics, stimulation of Cdc42 activity had the opposite effect on MT-dynamics than Diaph2. While Diaph2 decreased, stimulation of Cdc42 increased MT-stability, independent of the presence (control cells) or absence (Diaph2-depleted cells) of Diaph2 (see Fig. 4).

In order to substantiate our conclusion that Diaph2 increases MT-dynamics independent of Cdc42 activity, the effect of Diaph2 in cell-free reconstitution assays was analyzed, employing bacterially produced Diaph2 and purified tubulin as well as the isolated FH2 domain as control. Intriguingly, a MT-turbidity assay revealed that Diaph2 increased the velocity of microtubule polymerization 10-fold stronger than the FH2-domain and, in contrast to the FH2-domain, nucleated MT-polymerization. The finding that Diaph2 stimulates MT-polymerization was confirmed by the use of rhodamine-labeled MTs, enabling to directly visualize formation of long MTs (see Fig. 6A, upper panel). These results support our conclusion that Diaph2 increases MT-dynamics in a confirmation where its actin nucleating activity is auto-inhibited.

In addition to examining the effect of Diaph2 on MT-dynamics, we analyzed if Diaph2 may alter the density of MTs, employing taxol treated rhodamine labeled MTs. We found that Diaph2 and the FH2-domain increased MT-density, most likely by binding as dimer to adjacent MT-bundles. However, this effect seems to be weak because it was not possible to spin down these MT-assemblies by low centrifugation (see Fig. 7F).

Our result, showing a stronger effect of Diaph2 on MT-polymerization relative to the FH2-domain, strongly indicated the existence of a second MT-binding domain inside the Diaph2 molecule. Indeed, a Diaph2-mutant lacking the FH2-domain (ΔFH2) stimulated MT-polymerization in a similar manner as the FH2-domain. However, different from the FH2-domain and full length Diaph2, the ΔFH2-mutant did not increase the density of taxol-stabilized MTs and did not protect against cold-induced MT-polymerization. These data indicate that the ΔFH2-mutant does not bind to adjacent MT-bundles and does not stabilize MTs. It is thus tempting to speculate that via the FH2 domain Diaph2 dimerizes and additionally weakly binds to adjacent MTs, resulting in stabilization of MTs. The ΔFH2-mutant also should form homodimers via the DD-domain (see Fig. 7A), but by this domain it cannot bind to MTs. This explains why the ΔFH2- mutant increases MT-polymerization but not MT-density.

Based on this model, we hypothesize that Diaph2 binds to αβ-tubulin dimers or to short MTs via the second MT-binding domain to recruit them to growing MTs and thereby increases MT-polymerization, while the MT-stabilizing effect of Diaph2 seems to be mediated through the dimeric FH2 domain by mediating the association of adjacent MTs. According to this model, Diaph2 would nucleate MTs by the second MT-binding domain and decreases catastrophe via the FH2 domain. However, in order to precisely elucidate the mechanism how Diaph2 increases MT-polymerization and density, identification of the second Diaph2-MT-binding domain in future experiments is required. A good candidate would be the basic region of a fragment C-terminally of the DAD-domain (aa 1048 to 1101) as it has been shown that in mDia1 and mDia2, this region increases the affinity of the FH1/FH2-domain to MTs^1^. Furthermore, the fragment C-terminally of the GBD domain (aa 156–455) has been shown to be essential for localization of mDia1 to spindle MTs^6^. Thus, also this fragment is likely to bind to MTs.

The *in vitro* data together with our cellular data lead us to the conclusion that independent of Cdc42-stimulation, Diaph2 increases spindle-MT dynamics near the spindle pole, by promoting MT-polymerization. Since Cheng *et al*.^2^ revealed that Diaph2-controlled spindle-MT-dynamics depend on the presence of EB1, we assume that Diaph2 binds to plus-ends of MTs, thereby promoting MT-polymerization of spindle MTs towards the kinetochore. Our result, showing that depletion of Diaph2 results in activation of the checkpoint protein BubR1, which recognizes failures in spindle attachment to the kinetochore, supports this assumption.

## Conclusions

We present here a new mechanism of how a formin controls MT-dynamics. Our data show that Diaph2 controls MT-dynamics even in its auto-inhibited form and outline a specific role of Diaph2 in the regulation of spindle MT-dynamics. Furthermore, we reveal that, in addition to the FH2-domain, a domain outside the FH2-domain is involved in Diaph2-controlled MT-dynamics. Future elucidation of this additional MT-binding domain will help to fully understand the mechanism of Diaph2-formin controlled MT-dynamics in spindle fibers.

## Supplementary information

Supplementary Information
