# Supplementary material for: The formin Drosophila homologue of Diaphanous2 (Diaph2) controls microtubule dynamics in colorectal cancer cells independent of its FH2-domain
Source: Sci Rep. 2019 Mar 29;9:5352. doi: 10.1038/s41598-019-41731-y (PMC6441084; doi:10.1038/s41598-019-41731-y)
Supplement: Supplementary file 1 — Supplementary Information [file 41598_2019_41731_MOESM1_ESM.docx]

**The formin Drosophila homologue of Diaphanous2 (DIAPH2) controls microtubule dynamics in colorectal cancer cells independent of its FH2-domain**

Saskia S Grueb^1^, Stefanie Muhs^1^, Yannes Popp^1^, Sebastian Schmitt^2^, Matthias Geyer^2^, Yuan-Na Lin^1,3^ & Sabine Windhorst^1^

^1^Department of Biochemistry and Signal Transduction, University Medical Center Hamburg-Eppendorf Martinistrasse 52, D-20246 Hamburg, Germany

^2^Institute of Structural Biology, University of Bonn, Sigmund-Freud-Str. 25, D-53127 Bonn, Germany

^3^Department of General, Visceral and Thoracic Surgery, University Medical Center Hamburg-Eppendorf Martinistrasse 52, 52 D-20246 Hamburg, Germany

**Address correspondence to:**

Sabine Windhorst

Department of Biochemistry and Signal Transduction,

University Medical Center Hamburg-Eppendorf,

Martinistrasse 52,

D-20246 Hamburg

Germany

Tel. +49-40-7410-56341

Fax +49-40-7410-56818

E-Mail: [s.windhorst@uke.uni-hamburg.de](mailto:s.windhorst@uke.uni-hamburg.de)

**Supplementary Data**

**
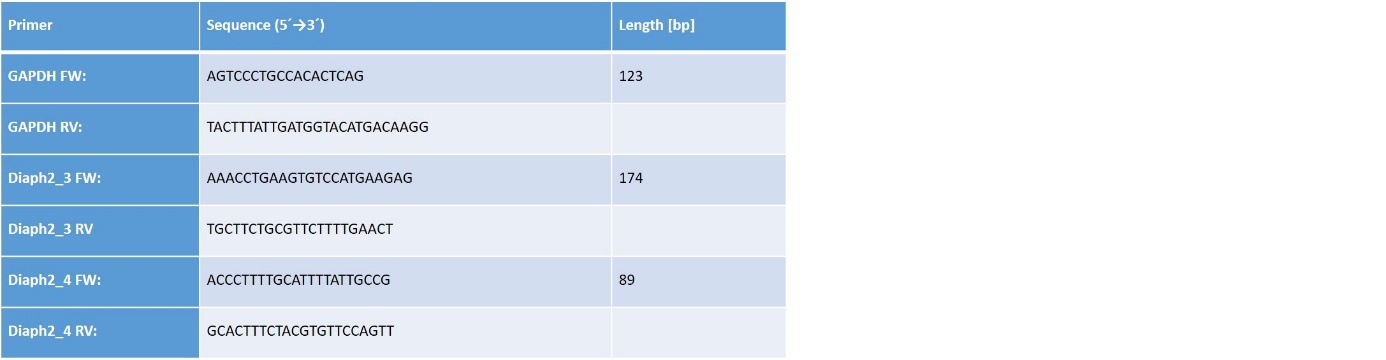
**

**Table S1. Oligonucleotides used to analyze DIAPH2 mRNA levels in HT29 cells.**


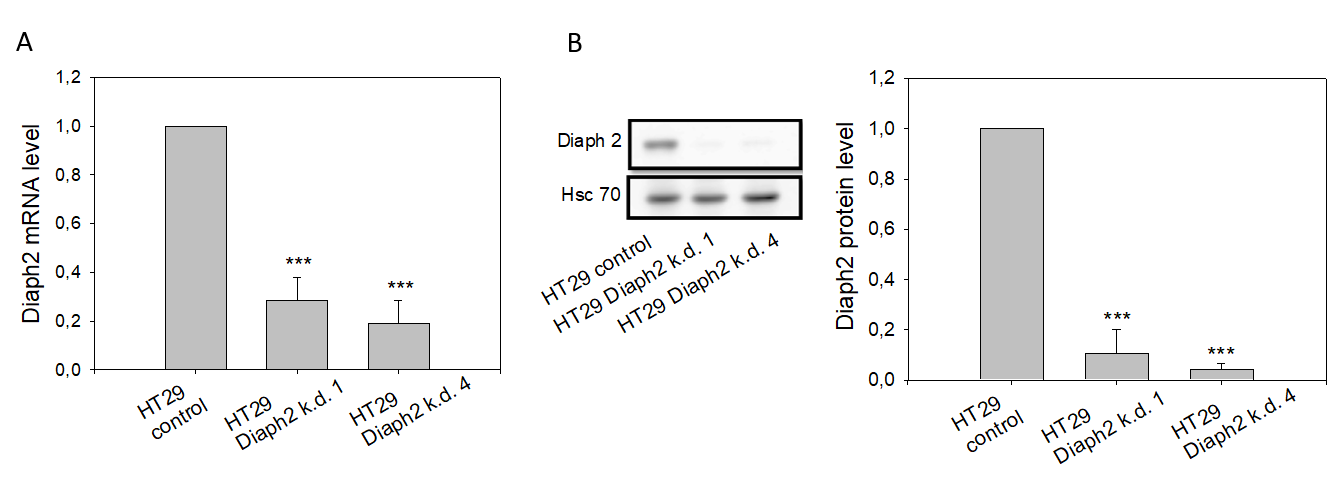


**Figure S1. DIAPH2 depletion in HT29 cells.** Cell lysates of four different time points from control and anti-DIAPH2 shRNA treated cells were analyzed by real-time PCR **(A)** or by Western blotting **(B).** Shown are mean values + SD of three different cell lysates. Normal distribution of band intensity values and qPCR values was assumed and evaluated with One-Way ANOVA and Bonferroni’s multiple comparison test, ***p<0.001.

**
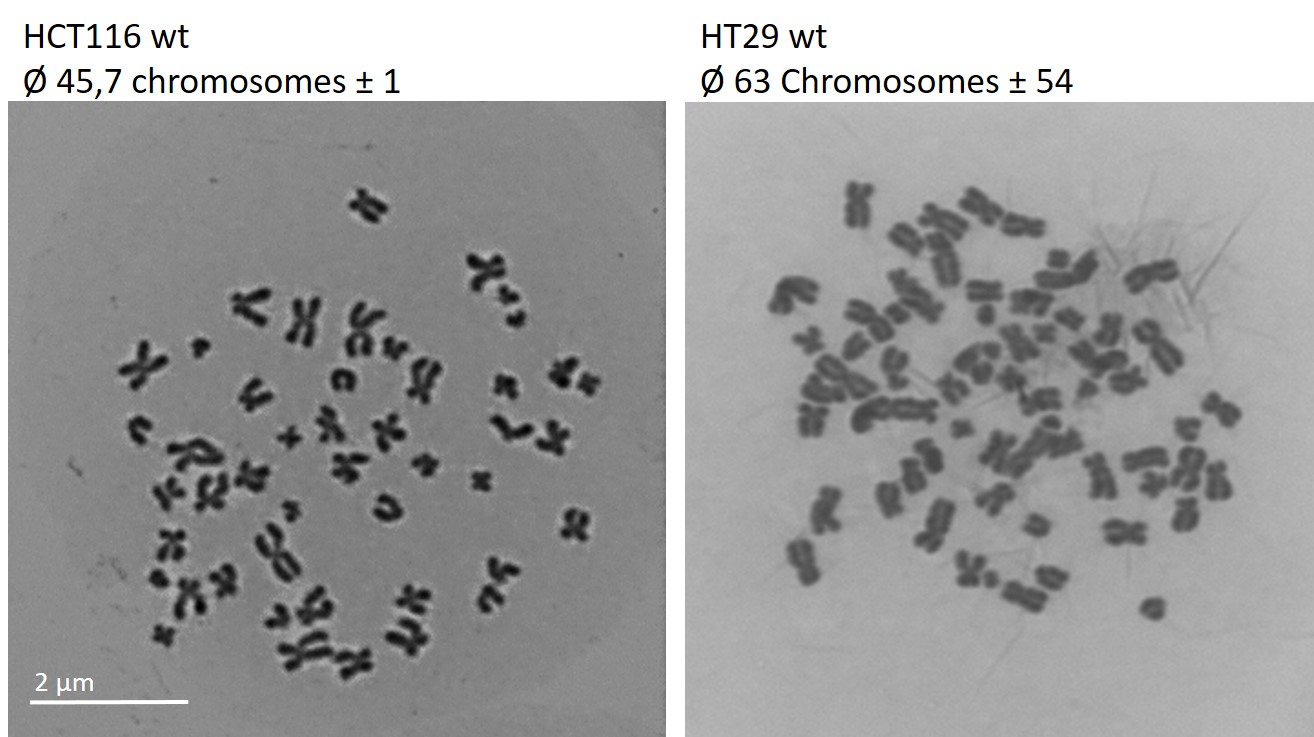
**

**Figure S2. Chromosomes from chromosomal stable (HCT116) and chromosomal instable (HT29) cells.** Chromosomes were prepared from metaphase cells and stained with Giemsa solution. Shown is one representative preparation from HCT116- and HT29 cells.

**
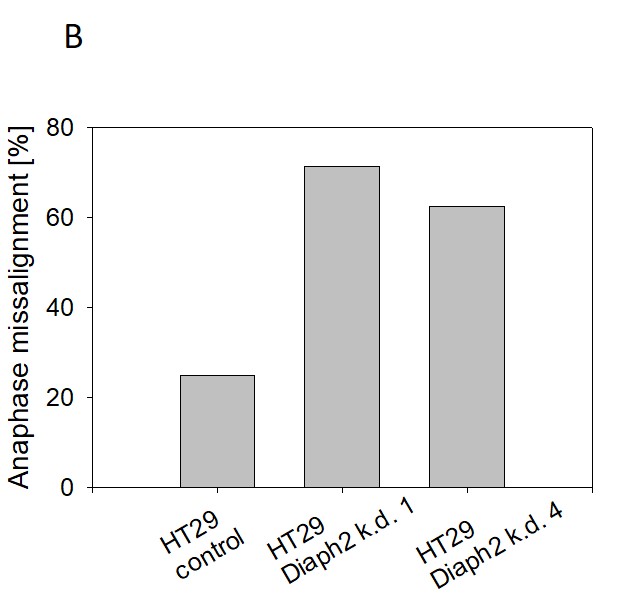

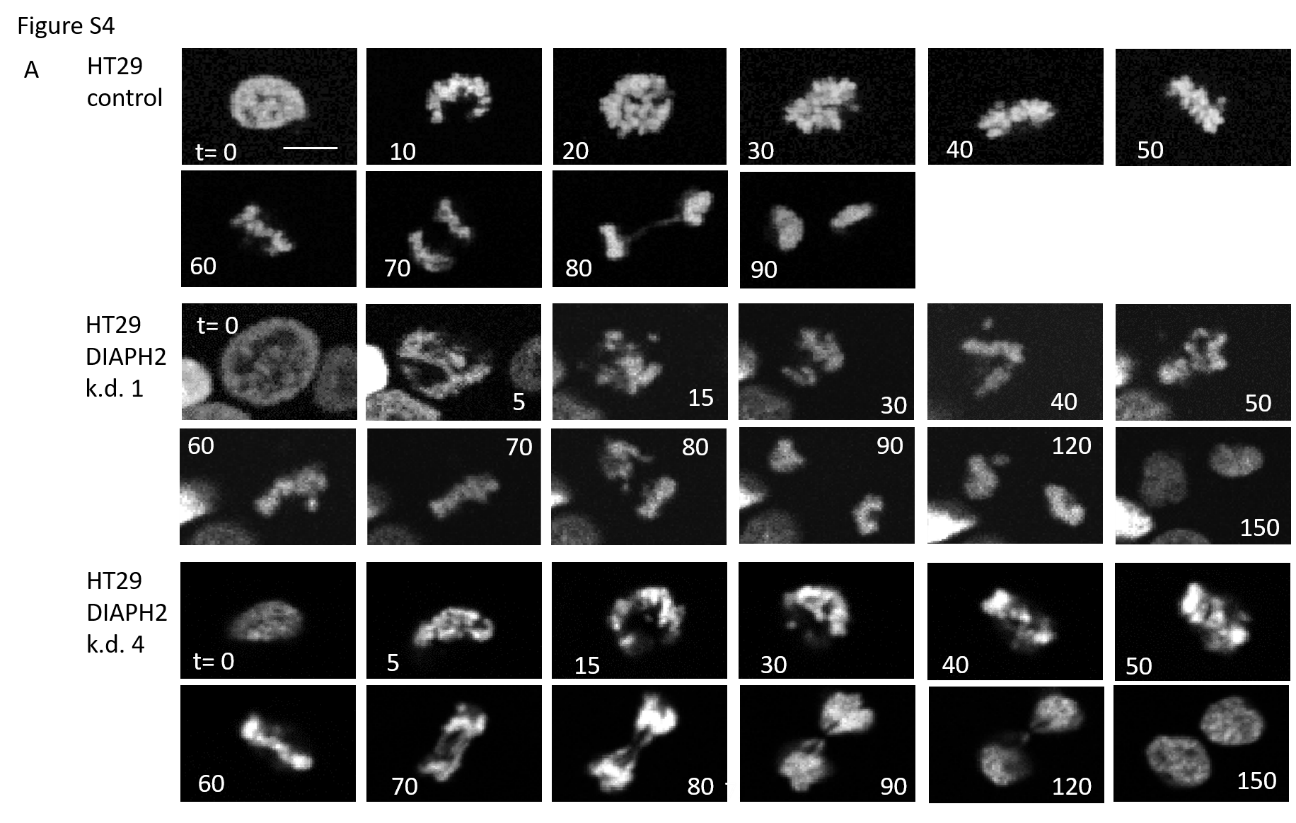
**

**Figure S3. M-phase progression in control and DIAPH2-depleted cells.** HT29 cells were transfected with a vector encoding for pH2B_EYFP and 12 h after transfection pH2B_EYFP fluorescence was monitored by live cell imaging. Shown are representative images from one analysis. Time is given in minutes. Scale bar represents 10 µm. **(B)** Chromosomal alignment was analyzed from anaphase cells shown in (A) as well from three further cells and the percentage of misaligned chromosomes was calculated.

**
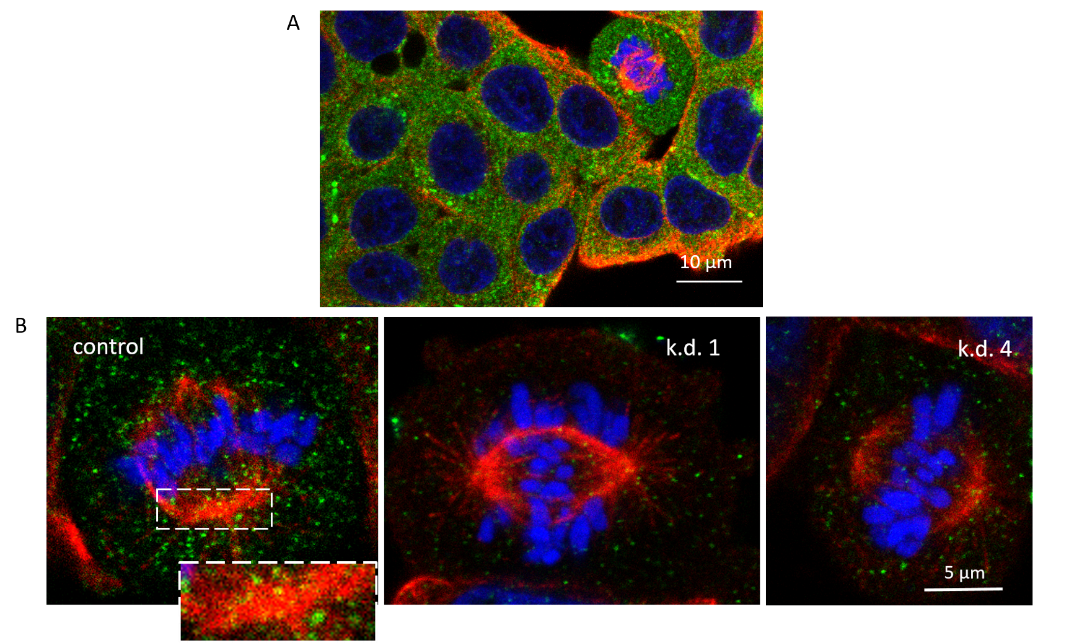
**

**
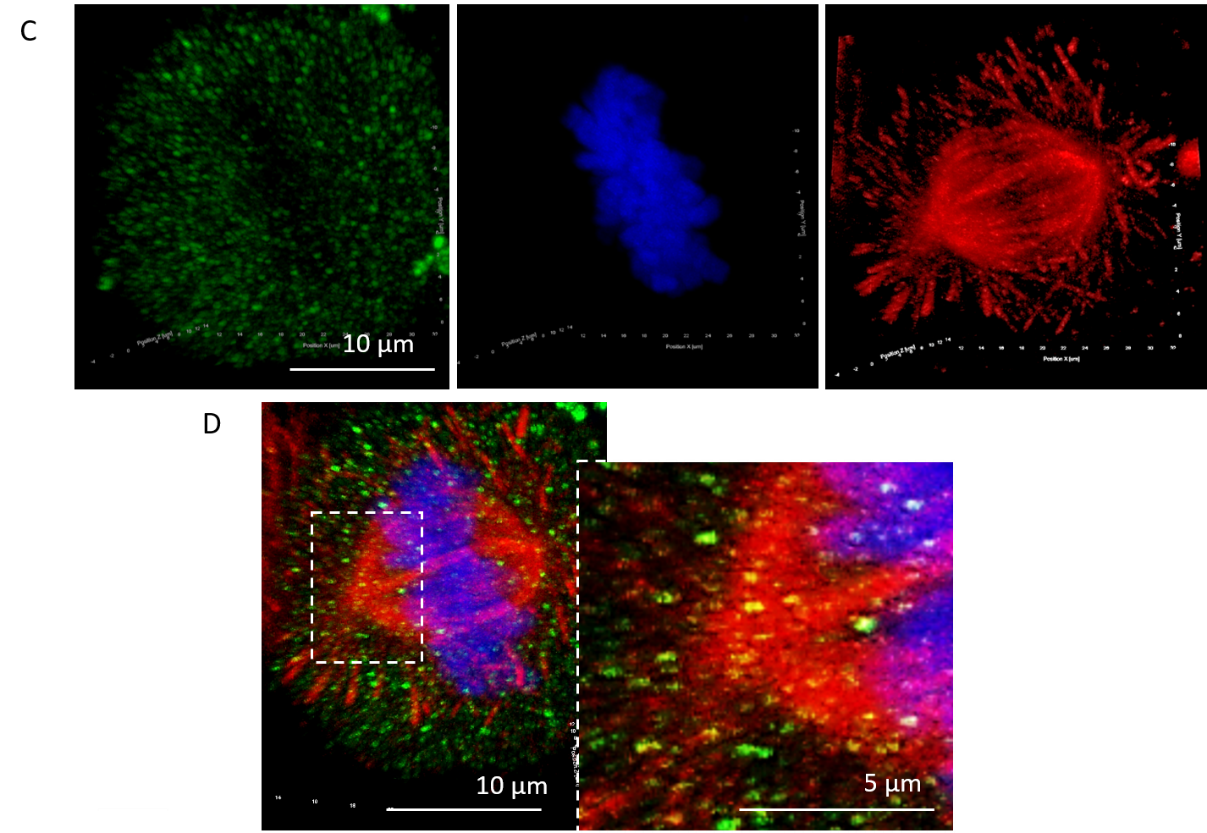
**

**Figure S4. Localization of DIAPH2 in HT29 cells. (A)** Control HT29 cells were stained with antibodies against DIAPH2 (green) and ß-tubulin (red) coupled with secondary Alexa-fluor antibodies and the nuclei were stained with DAPI. **(B)** The same treatment of HT29 control and DIAPH2 depleted cells (k.d. 1 and k.d. 4) as shown above to evaluate the specificity of the anti-DIAPH2 antibody. **(C, D)** 3D-reconstructios of DIAPH2 and MT-stained HT29 cells to calculate the co-localization index. (D) shows merged images including magnification.

**
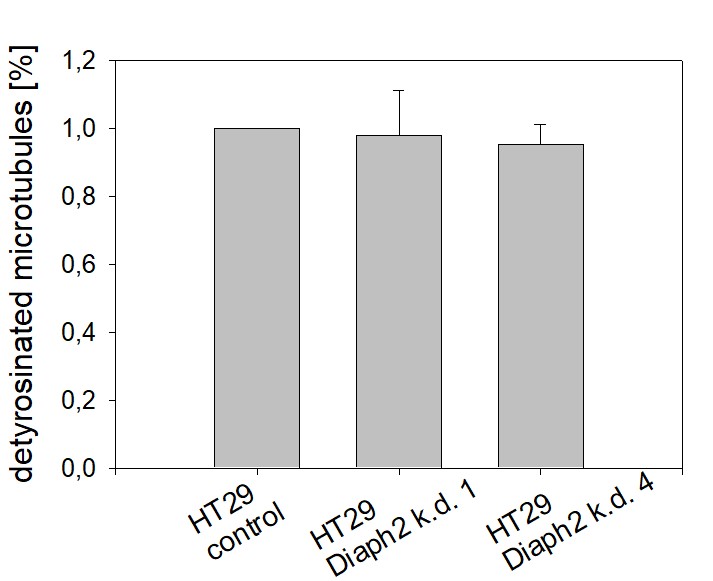
**

**Figure S5. Effect of DIAPH2 on MT-dynamics.** Cell lysates from control and DIAPH2-depleted cells were analyzed by Western blotting for the level of detyrosinated MTs as well as for ß-tubulin. Shown is the mean + SD ratio of detyr/panMTs obtained by 3 different experiments. Normal distribution of band intensity values and qPCR values was assumed and evaluated with One-Way ANOVA and Bonferroni’s multiple comparison test, values were found as not significantly altered.

**
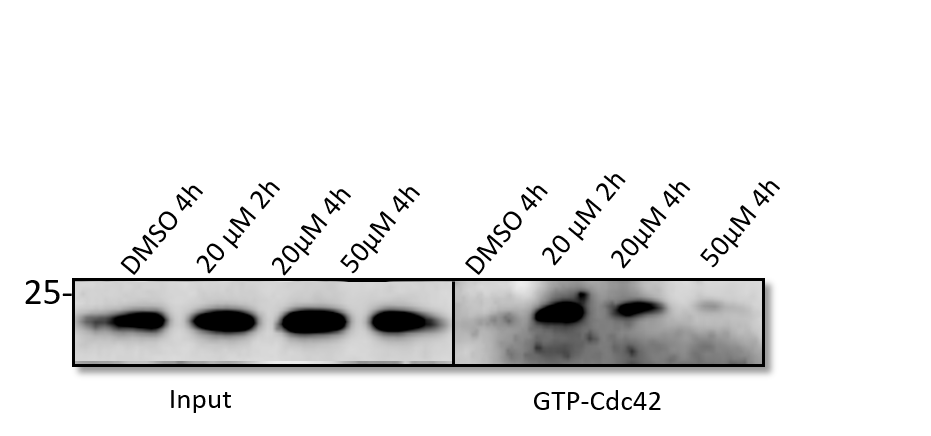
**

**Figure S6. Cdc42 activity in ML141** **treated cells.** Control HT29 cells were treated with 20 µM or with 40 µM ML141 for 2 h or 4 h. DMSO served as vehicle control. GTP‑bound Cdc42 was analyzed by PAK pulldown and Western blotting. Shown is one representative Western blot out of three.

**
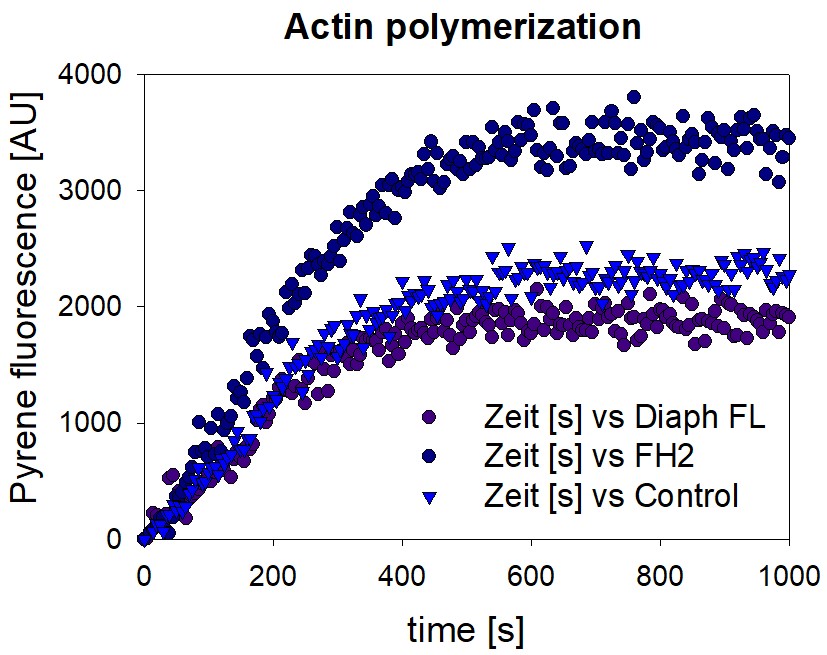
**

**Figure S7. Actin nucleating activity of FL-DIAPH2 and the FH2-domain.** Actin polymerization activity of FL-DIAPH2, the FH2-domain or GST (Control) was measured by the pyrene actin polymerization assay. Shown is one representative measurement out of three.

**
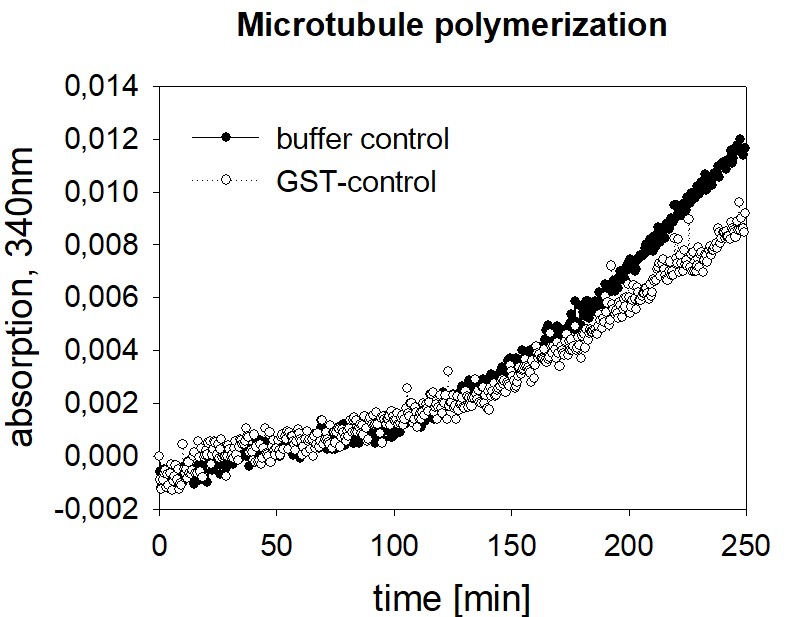
**

**Figure S8. Effect of GST on MT-polymerization.** The effect of GST or buffer was compared in terms of MT-polymerization of non-labeled, non-taxol treated MTs. Shown is one representative measurement out of three.
